# Nonlinear relationship between sleep midpoint and osteoarthritis: a cross-sectional study in US adults

**DOI:** 10.1007/s10067-025-07918-7

**Published:** 2026-01-17

**Authors:** Haifeng Yang, Weibin Liu, Chunqing Xiao, Yuanyuan Han, Xiaomin Lin, Yanping Wu

**Affiliations:** 1 Department of Arthroplasty, Ganzhou Hospital of Traditional Chinese Medicine, Ganzhou, Jiangxi China; 2 Department of Cardiology, Ganzhou Hospital-Nanfang Hospital, Southern Medical University (Ganzhou People’s Hospital), Ganzhou, Jiangxi China; 3 Medical Imaging Department, First Clinical Medical College of Gannan Medical University, Jiangxi, China; 4 Department of Dermatology, Ganzhou Women and Children’s Health Care Hospital, Jiangxi, China; 5 Department of Psychiatry, Ganzhou Hospital-Nanfang Hospital, Southern Medical University (Ganzhou People’s Hospital), Ganzhou, Jiangxi China

**Keywords:** Circadian rhythm, NHANES, Nonlinear association, Osteoarthritis, Sleep midpoint

## Abstract

**Background:**

Osteoarthritis (OA) is a common chronic degenerative joint disease globally. While sleep-related factors are linked to its pathogenesis, the specific association and nonlinear characteristics between sleep midpoint (the midpoint of the sleep cycle) and OA remain unclear.

**Methods:**

Data for this study were obtained from the National Health and Nutrition Examination Survey (NHANES 2015–2020). Sleep midpoint was calculated from self-reported weekday bedtime and wake-up time. Restricted cubic spline (RCS) models analyzed the relationship between sleep midpoint and OA, adjusted for demographic, lifestyle, and clinical covariates. Subgroup analyses and propensity score matching verified result robustness.

**Results:**

A total of 7640 participants were included in this study, of whom 977 had OA. RCS revealed a “J-shaped” association with an inflection point at 2:30 AM. No significant association was found for sleep midpoint < 2:30 AM (OR = 0.95, 95% CI = 0.88–1.02). For sleep midpoint ≥ 2:30 AM, each 30-min delay was associated with an 8% increased OA prevalence (OR = 1.08, 95%CI = 1.00–1.18). Subgroup and matched analyses confirmed robustness.

**Conclusions:**

Our study results indicate that in a representative sample of US adults, a delayed sleep midpoint (≥ 2:30 AM) is independently associated with OA, suggesting that a delayed sleep midpoint beyond 2:30 AM is linked to a higher prevalence of OA. This highlights the potential role of sleep midpoint in OA prevention and management.

**Table:** 

**Key Points** • *Sleep midpoint shows a nonlinear association with osteoarthritis prevalence, with an inflection around 02:30 AM.* • *After approximately 02:30 AM, each 30-min delay in sleep midpoint is associated with ~ 8% higher adjusted odds of osteoarthritis, independent of demographics, lifestyle factors, comorbidities, and sleep duration.* • *Results are consistent across subgroups and remain robust in propensity score–matched and sensitivity analyses using survey weighted, nationally representative US data.* • *Sleep timing is modifiable, indicating a practical target for risk reduction that warrants prospective and interventional evaluation.*

**Supplementary Information:**

The online version contains supplementary material available at 10.1007/s10067-025-07918-7.

## Introduction

Osteoarthritis (OA), the most common chronic degenerative joint disease worldwide, is characterized by core pathological features including articular cartilage degeneration, osteophyte formation, and synovial inflammation, which severely impair patients’ joint function and quality oflife [1, 2]. Statistics indicate that approximately 595 million people worldwide are affected by it [3]. With the acceleration of population aging and the growing severity of obesity, the prevalence of OA continues to rise, becoming a heavy burden on global healthcare systems [4]. Therefore, an in-depth elaboration of its pathogenesis and related influencing factors is of great significance for formulating effective prevention and treatment strategies.

Sleep midpoint refers to the midpoint time of an individual’s sleep cycle, reflecting the temporal distribution of sleep [5]. Abnormal sleep midpoint (either excessively early or late) has been confirmed to be closely linked to the risk of multisystem diseases such as metabolic disorders, hypertension, and depression [6, 7]. A study by Zhihan Zhai et al. demonstrated that either excessively early or late sleep midpoint may increase the risk of type 2 diabetes [8]; it exhibits a nonlinear association with depressive symptoms—when sleep midpoint is between 2:18 AM and 6:30 AM, each 1-h delay increases the risk of depressive symptoms by 20% (OR = 1.20, 95% CI 1.08–1.33) [9]. Additionally, irregular sleep midpoint significantly elevates the risk of hypertension [10]. Existing studies have found that delayed or fluctuating sleep midpoint can promote the development of OA through pathways involving inflammatory responses and metabolic disorders by reducing physical activity levels, increasing inflammatory factors, and inducing insulin resistance [11–17].

However, the specific association and potential dose–response relationship between sleep midpoint and OA remain unclear. Previous studies have mostly focused on the association between sleep duration and OA [18, 19]. As a key indicator reflecting sleep quality, the nonlinear association and threshold effect between sleep midpoint and OA have not been explored. In view of this, this study utilized the large nationally representative dataset of the National Health and Nutrition Examination Survey (NHANES) to systematically investigate the impact of sleep midpoint on the prevalence of OA, aiming to provide a new theoretical basis and practical directions for the early prevention and intervention of OA.

## Materials and methods

### Study population

This study used data from the NHANES, a survey conducted by the National Center for Health Statistics (NCHS) to assess the health and nutritional status of the population. The NHANES protocol was approved by the NCHS Institutional Review Board, and all participants provided written informed consent. The study was conducted in accordance with relevant guidelines and regulations (further information is available on the CDC website).

**Fig. 1:**
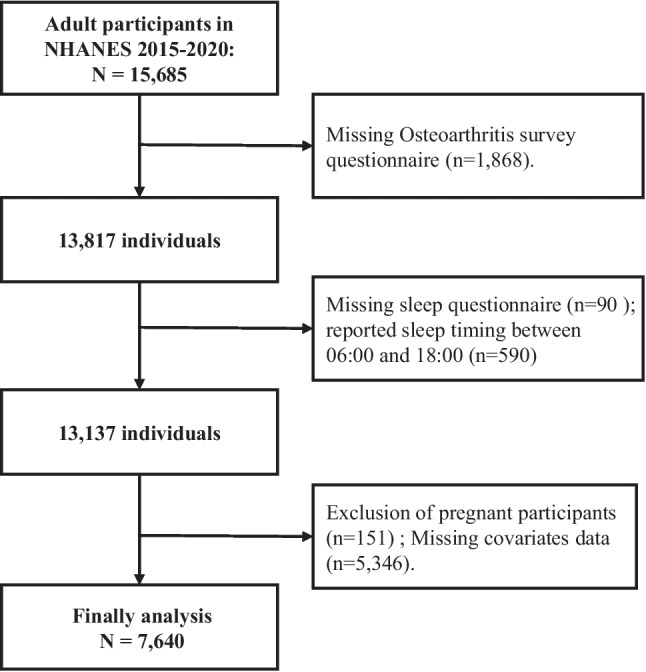
Flowchart showing the number of NHANES participants included in the current analyses. Abbreviations: NHANES, National Health and Nutrition Examination Survey; PSM, propensity score matching

A total of 15,685 adults from the 2015–2020 NHANES surveys were included, with self-reported nighttime bedtime and regular wake-up time collected. Exclusion criteria were as follows: (1) missing OA diagnosis information (*n* = 1868); (2) missing sleep duration data (*n* = 90); (3) sleep timing between 06:00 and 18:00 (daytime sleep) (*n* = 590) excluded because the study focuses on the association between nighttime sleep midpoint and circadian rhythms, while the temporal distribution of daytime sleep has weak relevance to physiological rhythms [20]; (4) pregnant participants (*n* = 151); (5) missing data on relevant covariates (*n* = 5346) (Supplementary Fig. 1). Finally, 7640 participants were included (Fig. 1).

## Sleep assessment

In accordance with recommendations from the National Sleep Foundation, nighttime sleep duration was calculated as the interval between sleep onset and wake-up time. Sleep midpoint was computed based on self-reported bedtime and wake-up time, defined as the midpoint between falling asleep and waking up [21, 22]. Information on bedtime and wake-up time was obtained from responses to the following questions: “What time do you usually go to bed on weekdays/workdays?” and “What time do you usually wake up on weekdays/workdays?”.

## Definition of OA

OA diagnosis information was derived from the NHANES physical condition questionnaire: participants were asked, “Has a doctor or other health professional ever told you that you have arthritis?” If the answer was “yes” a follow-up question was asked: “What type of arthritis was it?” Participants who self-reported “OA or degenerative arthritis” were classified as OA patients (including all anatomical sites); those reporting “rheumatoid arthritis,” “psoriatic arthritis,” or “other” were categorized as non-OA individuals. Previous studies have confirmed good consistency between self-reported OA in NHANES and clinical diagnosis [23].

## Covariates

The following potential covariates were included: (1) demographic characteristics: age, sex, race/ethnicity, family poverty, educational level (≤ high school vs. > high school); (2) lifestyle factors: alcohol consumption status (never/current), smoking status (nonsmoker/former smoker/current smoker), sedentary time, and physical activity; and (3) clinical indicators: hypertension, diabetes, stroke, coronary artery disease, hyperlipidemia, hyperuricemia (defined as serum uric acid ≥ 7.0 mg/dL in men, ≥ 6.0 mg/dL in women, or current use of urate-lowering medications), waist circumference, and BMI; (4) other factors: sleep duration, sleep quality (frequency of difficulty falling/staying asleep or excessive sleep in the past 2 weeks), and Healthy Eating Index (HEI-2015) (calculated from NHANES dietary questionnaires based on adherence to 9 food groups, with a total score of 0–100) [24].

## Statistical analysis

To ensure national representativeness, sample-specific weights, stratification, and clustering were incorporated into the analysis. All analyses were performed using R (version 4.4.1, Vienna, Austria). A two-sided *p* < 0.05 was considered statistically significant.

Continuous variables were expressed as weighted mean ± standard deviation (SD) and categorical variables as frequency and weighted percentage. Wilcoxon rank-sum test was used for continuous variables, and weighted chi-square test for categorical variables to determine statistical differences between groups. Sample-weighted logistic regression models were applied to calculate odds ratios (ORs) and 95% confidence intervals (CIs) and assess the association between sleep midpoint and OA. Model 1 was adjusted for age, sex, and race/ethnicity. Model 2 was adjusted for age, sex, race/ethnicity, educational levels, family poverty, marital status, smoking status, and alcohol consumption. Model 3 was adjusted for variables in Model 2 plus hypertension, diabetes, coronary artery disease, stroke, hyperlipidemia, hyperuricemia, waist circumference, BMI, Healthy Eating Index (score), sedentary time (minutes), and sleep disturbance. Restricted cubic spline (RCS) models were used to explore potential dose–response associations between sleep midpoint and OA, with three knots set at the 10th, 50th, and 90th percentiles. Additionally, the threshold effect of sleep midpoint on depressive symptoms was further investigated. Likelihood ratio tests comparing single-line and piecewise regression models were used to determine the presence of thresholds. To further evaluate the reliability of the association between sleep midpoint and OA, subgroup analyses were performed by age (< 65 years vs. ≥ 65 years), sex, race/ethnicity (non-Hispanic White vs. other races), educational level, family poverty, alcohol consumption, smoking status, and sleep disturbance to investigate correlations in each subgroup. Interaction tests were used to assess the consistency of associations across subgroups.

To control for potential confounding bias, propensity score greedy matching was performed using a 3:1 ratio, with the caliper width set to 0.2 times the standard deviation of the logit of the propensity score, aiming to achieve balance in the distribution of covariates between groups. Matching variables included age, sex, race/ethnicity, educational levels, family poverty, marital status, smoking status, alcohol consumption, hypertension, diabetes, coronary artery disease, stroke, hyperlipidemia, hyperuricemia, waist circumference, BMI, HEI-2015, sedentary time, and sleep disturbance. Covariate balance after matching was verified by standardized mean difference (SMD < 0.1).

## Results

### Participant clinical characteristics

A total of 7640 participants were included, with 977 having OA (weighted prevalence 14.24%). Females accounted for 3891 participants (51.22%). The weighted mean age of participants was 46.89 years, and the mean sleep duration was 7.62 h. Significant differences were observed between OA and non-OA groups in age, sex, race/ethnicity, family poverty, marital status, smoking status, alcohol consumption, hypertension, diabetes, coronary artery disease, stroke, hyperlipidemia, hyperuricemia, waist circumference, BMI, HEI-2015, and sleep disturbance (Table 1). Clinical characteristics of matched participants are shown in Supplementary Table 1.

**Table 1:** Comparison of baseline characteristics of the study population before and after matching from NHANES 2007–2020

	Osteoarthritis			
Characteristic	Total	No	Yes	*p*value
**N (%)**	7640	6663 (85.76)	977 (14.24)	
**Age (years),** mean (95% CI)	46.89 (0.50)	44.40 (0.45)	61.90 (0.45)	< 0.01
**Sex**				< 0.01
Male	3749 (48.78)	3409 (51.64)	340 (31.60)	
Female	3891 (51.22)	3254 (48.36)	637 (68.40)	
**Races/ethnicity**				< 0.01
Non-Hispanic White	2848 (67.53)	2300 (65.11)	548 (82.16)	
Non-Hispanic Black	1629 (9.28)	1468 (9.88)	161 (5.68)	
Mexican American	1136 (8.31)	1048 (9.21)	88 (2.89)	
Other race	2027 (14.88)	1847 (15.81)	180 (9.28)	
**Educations levels**				0.43
Below high school	1304 (9.50)	1165 (9.75)	139 (8.00)	
High school	1682 (22.94)	1469 (22.77)	213 (23.96)	
College/above	4654 (67.55)	4029 (67.47)	625 (68.04)	
**Marital status**				< 0.01
Widowed or divorced or separated	1533 (16.62)	1204 (14.48)	329 (29.56)	
Never married	1435 (17.92)	1370 (20.10)	65 (4.76)	
Married or living with partner	4672 (65.46)	4089 (65.42)	583 (65.69)	
**Family poverty**				0.02
0–1.3	2065 (16.81)	1846 (17.35)	219 (13.57)	
1.3–3.5	2985 (34.64)	2613 (35.02)	372 (32.34)	
> 3.5	2590 (48.55)	2204 (47.63)	386 (54.09)	
**Smoking status**				< 0.01
Never	4493 (58.29)	4000 (59.66)	493 (50.03)	
Former	1769 (25.55)	1441 (23.88)	328 (35.64)	
Now	1378 (16.16)	1222 (16.46)	156 (14.33)	
**Alcohol consumption**				< 0.01
Never	962 (8.86)	867 (9.23)	95 (6.67)	
Former	539 (5.48)	440 (4.94)	99 (8.76)	
Now	6139 (85.66)	5356 (85.84)	783 (84.56)	
**Medical history**				
Hypertension	4091 (48.65)	3367 (45.26)	724 (69.10)	< 0.01
Diabetes	1117 (10.92)	886 (9.57)	231 (19.04)	< 0.01
Stroke	269 (2.52)	197 (1.82)	72 (6.73)	< 0.01
Coronary artery disease	681 (7.14)	492 (5.37)	189 (17.82)	< 0.01
Hyperlipidemia	5166 (66.60)	4367 (63.94)	799 (82.60)	< 0.01
Hyperuricemia	1478 (18.96)	1252 (18.50)	226 (21.75)	0.08
**Physical habit parameter**				
Waist circumference (cm)	100.30 (0.43)	99.53 (0.45)	104.98 (0.74)	< 0.01
BMI (kg/m^2^)	29.52 (0.17)	29.27 (0.18)	31.04 (0.31)	< 0.01
HEI-2015 (score)	53.16 (0.42)	52.73 (0.42)	55.74 (0.66)	< 0.01
Sedentary time (minutes)	372.88 (4.38)	371.15 (5.02)	383.35 (7.42)	0.2
**Sleep-related state**				
Sleep duration (hour)	7.62 (0.02)	7.60 (0.02)	7.74 (0.04)	< 0.01
Sleep midpoint				0.8
Early	3538 (46.89)	3117 (46.97)	421 (46.36)	
Late	4102 (53.11)	3546 (53.03)	556 (53.64)	
Sleep disturbance				< 0.01
Not at all	4703 (60.83)	4192 (61.89)	511 (54.39)	
Several days	1848 (25.03)	1583 (24.68)	265 (27.18)	
More than half the days	513 (7.54)	441 (7.65)	72 (6.87)	
Nearly every day	576 (6.60)	447 (5.78)	129 (11.56)	

*Numbers (*N*) in the table were unweighted. Percentages or means (95% CI) were estimated using US population weights

Race/ethnicity was determined using preferred terminology from the National Center for Health Statistics as non-Hispanic White, non-Hispanic Black, and Mexican American. Mexican–American individuals were oversampled rather than broader groups of individuals from Latin America. Other includes Asian, other Hispanic, Alaskan native, and multiracial individuals

Family poverty was presented as the ratio of family income to the federal poverty threshold, adjusted for household size, and is a measure of family income relative to poverty guidelines specific to the survey year

*N* number, *NHANES* National Health and Nutrition Examination Survey, *BMI* body mass index, *HEI* healthy eating index

## Relationship between sleep midpoint and OA

**Fig. 2:**
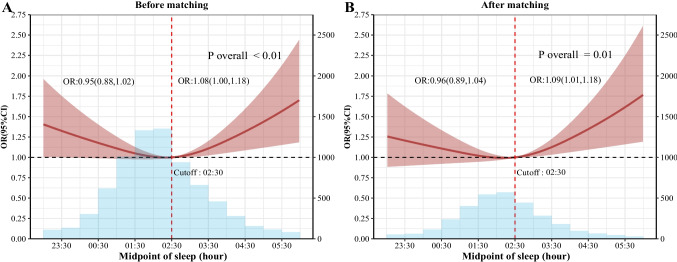
Adjusted spline curves analyze for the association of sleep midpoint with osteoarthritis disease among US adults. Solid lines were hazard ratios with 95%CI in shaded areas. Knot locations were the 10th, 50th, and 90th tertiles of sleep midpoint. All models were adjusted for age, sex, race/ethnicity, education levels, family poverty, marital status, smoking status, and alcohol consumption, hypertension, diabetes, coronary artery disease, stroke, hyperlipidemia, hyperuricemia, waist circumference, BMI, HEI-2015, sedentary time, sleep disturbance. Abbreviations: OR, odds ratio; CI, confidence interval; BMI, body mass index; HEI, healthy eating index

RCS analysis showed a significant “J-shaped” relationship between sleep midpoint and OA (Fig. 2): the curve was nearly horizontal when sleep midpoint < 2:30 AM, with 95% CI including 1.0 (no statistical significance); after sleep midpoint ≥ 2:30 AM, the curve rose steeply with increasing delay, and the lower bound of 95% CI > 1.0 (*p* < 0.05), confirming 2:30 AM as a clear inflection point. Based on threshold effect analysis, participants were divided into two groups using 2:30 AM as the cutoff: < 2:30 AM group and ≥ 2:30 AM group. In the unadjusted model, no significant association was found between sleep midpoint before 2:30 AM and OA (OR: 0.94, 95% CI: 0.88–1.00) (Model 1). No significant association was observed even after adjusting for all covariates (OR: 0.95, 95% CI: 0.88–1.02) (Model 3). However, a significant association was found in participants with sleep midpoint between 2:30 AM and 6:30 AM (OR: 1.1, 95% CI: 1.03–1.17) (Model 1). After full covariate adjustment (Model 3), each 30-min delay in sleep midpoint was associated with an 8% increase in OA prevalence (OR: 1.08, 95% CI: 1.00–1.18) (Table 2).

**Table 2:** Association between sleep midpoint and osteoarthritis disease

			Model 1		Model 2		Model 3	
Sleep duration turning point (K)	Participant	Event (%)	OR (95%CI)	*p*value	OR (95%CI)	*p* value	OR (95%CI)	*p* value
**Unmatching**								
Sleep midpoint after 6:30 AM and before 2:30 AM the next day	3538	421 (46.36)	0.94 (0.88, 1.00)	0.05	0.94 (0.88, 1.01)	0.08	0.95 (0.88, 1.02)	0.14
Sleep midpoint from 2:30 AM to 6:30 AM	4102	556 (53.64)	1.1 (1.03, 1.17)	< 0.01	1.1 (1.02, 1.18)	0.01	1.08 (1.00, 1.18)	0.04
*p* value for nonlinear			< 0.01		< 0.01		0.03	
**Matching**								
Sleep midpoint after 6:30 AM and before 2:30 AM the next day	1434	417 (46.93)	0.96 (0.9, 1.03)	0.22	0.96 (0.9, 1.03)	0.29	0.96 (0.89, 1.04)	0.29
Sleep midpoint from 2:30 AM to 6:30 AM	1733	543 (53.07)	1.1 (1.02, 1.18)	0.01	1.09 (1.01, 1.17)	0.02	1.09 (1.01, 1.18)	0.03
Log-likelihood ratio			0.01		0.03		0.05	

Model 1 was adjusted for age, sex, and race/ethnicity

Model 2 was adjusted for age, sex, race/ethnicity, education levels, family poverty, marital status, smoking status, and alcohol consumption

Model 3 was adjusted for the variables in Model 2 plus hypertension, diabetes, coronary artery disease, stroke, hyperlipidemia, hyperuricemia, waist circumference, BMI, HEI-2015, sedentary time, and sleep disturbance

*OR* odds ratio, *CI* confidence interval, *BMI* body mass index, *HEI* healthy eating index

## Subgroup analyses and propensity score matching analysis

To reduce confounding bias, 3167 participants were included after propensity score matching at a 1:3 ratio (960 in the OA group and 2207 in the non-OA group). The standardized mean difference (SMD) of all covariates was < 0.1, indicating good balance between groups (Supplementary Table 1 andSupplementary Fig. 2). In the matched cohort, RCS analysis showed a “J-shaped” relationship between sleep midpoint and OA, with trends consistent with the pre-matched cohort across Model 1, Model 2, and Model 3 (Fig. 2). Supplementary Table 2 shows the results of stratified analyses of the association between sleep midpoint and OA, stratified by age (< 65 years vs. ≥ 65 years), sex, race/ethnicity, educational levels, family poverty, alcohol consumption, smoking status, and sleep disturbance. No significant correlation was observed between sleep midpoint and OA in any specified subgroup. We also performed multiple interpolation analyses to reduce potential bias due to missing covariates; the results were consistent with the main analysis (Supplementary Table S3).

## Discussion

Based on cross-sectional analysis of the NHANES database, this study revealed a significant association between sleep midpoint and OA: when sleep midpoint exceeded 2:30 AM, each 30-min delay was associated with an 8% increase in OA prevalence, while no such association was observed before 2:30 AM. This finding suggests the potential link of delayed sleep midpoint disruption in OA pathology at the population level, providing a new perspective for research on OA pathogenesis and clinical prevention.

Previous studies have confirmed that delayed sleep midpoint can induce insulin resistance and glucose metabolism disorders, thereby increasing the prevalence of diabetes [25, 26]. In the field of cardiovascular diseases, abnormal sleep midpoint has also been proven to elevate the risk of diseases such as hypertension and coronary heart disease by increasing the levels of inflammatory factors and impairing vascular endothelial function [10, 27–29]. Our study found a positive correlation between delayed sleep midpoint and OA. Multiple previous studies have verified that sleep abnormalities are closely associated with OA. A cross-sectional study conducted in the Korean population revealed a positive correlation between abnormal sleep duration (including both insufficient and excessive sleep) and OA risk in middle-aged and elderly individuals [30]. Additionally, a study by Louis Jacob et al. pointed out that non-organic sleep disorders, hypersomnia, and sleep apnea are largely associated with a higher probability of developing OA [31]. Notably, shift workers are more prone to knee OA due to circadian rhythm disruption, a finding that is highly consistent with the results of our study [32]. As a crucial clinical indicator of sleep quality and circadian rhythm, delayed sleep midpoint not only expands the research scope of OA risk factors but also highlights the key role of healthy sleep habits in maintaining joint health. However, it is important to note that reduced sleep quality is a common symptom of knee OA [33]. Patients with OA may experience delayed sleep midpoint due to pain, while opioid administration for pain relief can improve their sleep quality [34], which reflects the potential bidirectional interaction between OA and sleep. The mechanisms underlying the association between delayed sleep midpoint and OA may be summarized as follows: Delayed sleep midpoint is frequently associated with circadian rhythm dysregulation, which can exacerbate joint inflammation by modulating the secretion of inflammatory factors [27]. In addition, delayed sleep midpoint may also perturb the secretion rhythms of melatonin and cortisol. Melatonin is known to exert chondroprotective effects, such that its aberrant secretion could accelerate joint degeneration [35]; conversely, dysregulated cortisol rhythms may further amplify inflammatory responses and exacerbate metabolic dysregulation [23]. Moreover, pain, genetic factors, and behavioral/lifestyle variables may contribute to the bidirectional interaction between delayed sleep midpoint and OA. Nevertheless, further longitudinal studies are needed to delineate the specific causal mechanisms governing these associations.

Notably, this study is the first to clarify the nonlinear association between sleep midpoint and OA: no association was observed when sleep midpoint was before 2:30 AM, but each 1-h delay between 2:30 AM and 6:30 AM was associated with an 8% increase in OA prevalence. The 2:30 AM sleep midpoint as a risk cutoff may have biological rationality. Physiologically, during sleep, the body undergoes a series of repair and regulatory activities, including hormone secretion, immune regulation, and cartilage metabolism [36, 37]. Previous studies have found that growth hormone secretion exhibits a significant circadian rhythm, with significantly higher nocturnal secretion than daytime. It has been observed that the peak concentration of growth hormone (GH) at night (23:00–03:00) is more pronounced than that in the daytime (11:00–15:00), and nocturnal measurements more accurately reflect the physiological secretion pool of GH [38–40]. Under normal sleep rhythms, growth hormone is secreted in large quantities during early nocturnal sleep, promoting chondrocyte proliferation and matrix synthesis [41]. Delayed sleep midpoint may lead to reduced or irregular growth hormone secretion, thereby affecting normal cartilage metabolism and repair, and increasing the risk of OA [41]. Additionally, delayed sleep midpoint may affect immune system regulation [42]. The immune system plays an important role in the development of OA, and the inflammatory response is one of the key pathological features of OA [43]. Normal sleep helps maintain immune system balance, while sleep rhythm disruption may lead to imbalances in immune cell activity and cytokine secretion, promoting the release of inflammatory factors such as interleukin-6 (IL-6) and tumor necrosis factor-α (TNF-α), triggering and exacerbating joint inflammation, thereby promoting the development of OA [44, 45].

The results of this study have important guiding significance for the clinical prevention and treatment of OA. First, clinicians should include sleep midpoint in sleep assessments when evaluating OA patients’ conditions. For patients with delayed sleep midpoint, vigilance should be raised regarding the increased prevalence of OA, and corresponding interventions should be implemented. Second, for patients with delayed sleep midpoint, sleep hygiene education and behavioral therapy can be used to help adjust their circadian rhythms, advancing sleep midpoint to before 2:30 AM, which may reduce the risk of OA or delay disease progression. Furthermore, for patients already diagnosed with OA, improving circadian rhythms and correcting delayed sleep midpoint may help alleviate symptoms and enhance treatment efficacy.

This study has certain limitations. First, the cross-sectional study design can only reveal an association between sleep midpoint and OA, and cannot establish a causal relationship. However, it is also important for us to note that pain associated with OA may lead to delayed sleep midpoint and sleep disturbances. To address this, prospective cohort studies or interventional studies will be needed in the future to further validate the causal relationship between sleep midpoint and OA, and to explore the underlying mechanisms. Second, data in the NHANES database may have certain measurement errors and information bias; for example, sleep midpoint assessment may rely on self-reporting by patients, leading to recall bias (For instance, individuals with long-term irregular sleep schedules may struggle to accurately recall their “typical bedtime”; alternatively, due to social desirability bias, they may overestimate their bedtime and underestimate their wake-up time, resulting in an earlier calculated sleep midpoint. In contrast, some middle-aged and elderly participants may experience memory decline, leading to blurred boundaries between pre-sleep activities (for example, watching TV or using electronic devices) and their actual bedtime, which further amplifies the error. OA diagnosis relies on self-reported “doctor-confirmed diagnosis history”). Third, while we adjusted for known potential confounders in our analyses, residual unmeasured confounders may still have influenced the results. For instance, factors such as depression, anxiety, shift work, analgesic use, physical activity intensity, and pain severity could have introduced bias into the observed association between sleep midpoint and OA. To address this limitation, future studies could enhance the robustness of findings by incorporating more comprehensive measurement of relevant variables and conducting additional sensitivity analyses to quantify the potential impact of unmeasured confounding. Fourth, the NHANES 2015–2020 database used in this study only collected overall information on “whether participants had received a doctor’s diagnosis of OA” via questionnaires and did not further subdivide the specific affected joint sites of OA (for example, knees, hips, and hands). This resulted in the inability to conduct site-stratified analyses to verify site-specific associations. Additionally, the results of this study are mainly applicable to the nighttime sleep pattern, while the daytime sleepers are still an important subgroup for future special research.

## Conclusion

In summary, the present study found a significant association between sleep midpoint and OA: A delayed sleep midpoint beyond 2:30 AM is associated with a higher prevalence of OA. This finding provides a new direction for OA research, and clinicians should pay attention to the potential role of sleep midpoint in OA.

## Supplementary Information

Below is the link to the electronic supplementary material.

Supplementary file 1 (PDF 487 KB)

## Data Availability

All data used in this study is available in the NHANES database: NHANES (National Health and Nutrition Examination Survey) homepage (cdc.gov).

## Abbreviations

NHANES: National Health and Nutrition Examination Survey
PSM: Propensity score matching
OR: Odds ratio
CI: Confidence interval
BMI: Body mass index
HEI: Healthy eating index

## Declarations

### Disclaimer

Y.W. is the guarantor of this work and, as such, has full access to all the data in the study and takes responsibility for the integrity of the data and the accuracy of the data analysis.
