# Supplementary material for: Nonlinear relationship between sleep midpoint and osteoarthritis: a cross-sectional study in US adults
Source: Clin Rheumatol. 2026 Jan 17;45(3):1877–86. doi: 10.1007/s10067-025-07918-7 (PMC12923419; doi:10.1007/s10067-025-07918-7)
Supplement: Supplementary file 1 — (PDF 487 KB) [file 10067_2025_7918_MOESM1_ESM.pdf]

Supplementary Online Content

Supplement Table 1. Comparison of Baseline Characteristics of the Study Population after Matching from NHANES 2007-2020.

Supplement Table 2. Association between Sleep Midpoint and Osteoarthritis in Stratified Analyses.

Supplementary Table 3. after Using Survey Impute for Multiple Interpolation.

Supplement Figure 1. Characteristics of the Missing Values of the Covariates.

Supplement Figure 2. Jitter Diagram of Propensity Score Matching.

This supplementary material has been provided by the authors to give readers additional information about their work.

Supplement Table 1. Comparison of Baseline Characteristics of the Study Population after Matching from NHANES 2007-2020.

Characteristic	Osteoarthritis			
	**Total**	**NO**	**Yes**	***P* value**
**N (%)**	3167	2207(71.39)	960(33.70)	
**Age (years),** mean (95% CI)	58.88(0.47)	57.51(0.55)	61.78(0.48)	< 0.01
**Sex**				0.36
Male	1240(38.24)	903(38.46)	337(32.00)	
Female	1927(66.85)	1304(61.54)	623(68.00)	
**Races/ethnicity**				0.65
Non-Hispanic White	1594(83.80)	1062(78.72)	532(81.90)	
Non-Hispanic Black	602(7.16)	441(7.29)	161(5.78)	
Mexican American	314(3.73)	226(3.83)	88(2.94)	
Other Race	657(10.41)	478(10.15)	179(9.38)	
**Educations Levels**				0.99
Below high school	470(7.85)	335(7.31)	135(7.82)	
High School	690(23.60)	480(21.70)	210(24.08)	
College/Above	2007(73.63)	1392(70.99)	615(68.11)	
**Marital status**				0.98
Widowed or divorced or separated	981(26.72)	662(23.80)	319(28.87)	
Never married	234(6.37)	170(6.67)	64(4.77)	
Married or living with partner	1952(72.00)	1375(69.53)	577(66.36)	
**Family poverty**				0.99
0-1.3	741(13.27)	524(12.12)	217(13.69)	
1.3-3.5	1202(33.34)	838(31.58)	364(32.04)	
>3.5	1224(58.48)	845(56.30)	379(54.27)	
**Smoking status**				0.92
Never	1618(53.79)	1131(51.72)	487(50.04)	
Former	1011(35.37)	689(32.67)	322(35.74)	
Now	538(15.93)	387(15.61)	151(14.22)	
**Alcohol consumption**				0.97
Never	324(7.39)	229(7.14)	95(6.80)	
Former	312(8.31)	214(7.50)	98(8.76)	
Now	2531(89.39)	1764(85.35)	767(84.44)	
**Medical history**				
Hypertension	2294(70.13)	1585(65.77)	709(68.77)	0.74
Diabetes	717(17.65)	494(16.02)	223(18.44)	0.93
Stroke	197(4.70)	128(3.51)	69(6.50)	0.78

Coronary artery disease	529(14.22)	347(11.75)	182(17.31)	0.97
Hyperlipidemia	2565(85.23)	1781(80.49)	784(82.39)	0.54
Hyperuricemia	743(22.89)	521(21.84)	222(21.68)	0.57
**Physical habit parameter**				
Waist circumference (cm)	104.58(0.49)	104.50(0.58)	104.75(0.75)	0.78
BMI (kg/m^2)	30.79(0.20)	30.74(0.25)	30.89(0.31)	0.7
HEI-2015 (score)	55.40(0.55)	55.21(0.62)	55.79(0.66)	0.4
Sedentary time (minutes)	385.09(4.99)	386.40(6.38)	382.30(7.38)	0.67
**Sleep-related state**				
Sleep duration (hour)	7.66(0.04)	7.62(0.05)	7.74(0.05)	0.04
Sleep midpoint				0.79
Early	1434(50.56)	1017(48.67)	417(46.93)	
Late	1733(54.53)	1190(51.33)	543(53.07)	
Sleep disturbance				0.96
Not at all	1719(57.77)	1211(55.01)	508(54.89)	
Several days	856(29.60)	596(28.61)	260(27.23)	
More than half the days	230(7.57)	161(7.50)	69(6.56)	
Nearly every day	362(10.15)	239(8.88)	123(11.32)	

***Numbers (N)** in the table were unweighted. Percentages or mean (95% CI) were estimated using US population weights.

Race/ethnicity was determined using preferred terminology from the National Center for Health Statistics as non-Hispanic White, non-Hispanic Black, and Mexican American. Mexican-American individuals were oversampled rather than broader groups of individuals from Latin America. Other include Asian, other Hispanic, Alaskan native, and multiracial individuals.

Family poverty was presented as the ratio of family income to the federal poverty threshold, adjusted for household size, and is a measure of family income relative to poverty guidelines specific to the survey year.

Abbreviations: N, number; BMI, body mass index; NHANES, National Health and Nutrition Examination Survey; BMI, body mass index; OA,Osteoarthritis;HEI, healthy eating index.

Supplement Table 2. Association between Sleep Midpoint and Osteoarthritis in Stratified Analyses.

	**Unmatching**				**Matching**			
Osteoarthritis	before 2:30 AM	after 2:30 AM	***P* value**	***P* for interaction**	before 2:30 AM	after 2:30 AM	***P* value**	***P* for interaction**
**Age group**				0.56				0.64
≤65 years	reference	0.95(0.69,1.31)	0.73		reference	0.95(0.67,1.34)	0.75	
>65 years	reference	1.11(0.74,1.67)	0.57		reference	1.09(0.71,1.66)	0.66	
**Sex**				0.03				0.01
Male	reference	0.71(0.44,1.12)	0.12		reference	0.67(0.40,1.14)	0.13	
Female	reference	1.23(0.95,1.60)	0.11		reference	1.30(0.98,1.71)	0.06	
**Race/ethnicity**				0.22				0.63
Non-Hispanic White	reference	0.94(0.73,1.22)	0.63		reference	0.98(0.76,1.26)	0.85	
Non-Hispanic Black	reference	1.03(0.70, 1.51)	0.87		reference	1.01(0.64,1.59)	0.96	
Mexican American	reference	1.33(0.71, 2.47)	0.28		reference	1.58(0.93, 2.68)	0.09	
Other Race	reference	1.17(0.70, 1.98)	0.52		reference	0.91(0.47, 1.78)	0.78	
**Education Level**				0.73				0.72
Below high school	reference	0.89(0.52,1.51)	0.63		reference	0.97(0.56,1.69)	0.91	
High School	reference	1.00(0.63,1.57)	1		reference	0.90(0.57,1.40)	0.61	
College/Above	reference	1.01(0.76,1.33)	0.96		reference	1.03(0.78,1.37)	0.8	
**Family poverty**				0.39				0.31
0-1.3	reference	0.96(0.62,1.47)	0.83		reference	0.91(0.65,1.28)	0.58	
1.3-3.5	reference	0.88(0.65,1.20)	0.39		reference	0.76(0.49,1.18)	0.2	
>3.5	reference	1.09(0.71,1.68)	0.67		reference	1.12(0.72,1.72)	0.59	
**Smoking status**				0.15				0.32

Never	reference	1.21(0.87,1.69)	0.24		reference	1.19(0.83,1.70)	0.31	
Former	reference	0.79(0.51,1.21)	0.25		reference	0.84(0.54,1.32)	0.42	
Now	reference	0.83(0.49, 1.42)	0.47		reference	0.85(0.50, 1.44)	0.51	
**Alcohol consumption**				0.13				0.09
Never	reference	1.90(1.06, 3.38)	0.03		reference	2.42(1.12, 5.24)	0.03	
Former	reference	0.87(0.54,1.39)	0.53		reference	0.87(0.52,1.45)	0.56	
Now	reference	0.96(0.74,1.24)	0.73		reference	0.97(0.76,1.25)	0.82	
**Sleep disturbance**				0.52				0.63
Not at all	reference	0.89(0.65,1.22)	0.44		reference	0.92(0.67,1.27)	0.61	
Several days	reference	1.11(0.61,2.00)	0.72		reference	1.11(0.62,1.99)	0.72	
More than half the days	reference	1.22(0.63, 2.38)	0.53		reference	1.09(0.50, 2.37)	0.81	
Nearly every day	reference	0.97(0.48, 1.95)	0.92		reference	1.10(0.51, 2.38)	0.79	

All models were adjusted for age, sex, race/ethnicity, education levels, family poverty, marital and smoking status, and alcohol consumption, hypertension, diabetes, coronary artery disease, stroke, hyperlipidemia, hyperuricemia, waist circumference, BMI, HEI-2015, sedentary time, sleep disturbance.

Abbreviations: OR, odds ratio; CI, confidence interval; BMI, body mass index; HEI, healthy eating index.

Supplementary Table 3. after Using Survey Impute for Multiple Interpolation.

	Model 1		Model 2		Model 3	
**Sleep duration turning point(K)**	**OR (95%CI)**	***P* value**	**OR (95%CI)**	***P* value**	**OR (95%CI)**	***P* value**
**Unmatching**						
Sleep midpoint after 6:30 AM and before 2:30 AM the next day	0.96(0.90,1.02)	0.17	0.96(0.90,1.02)	0.19	0.97(0.90,1.02)	0.26
Sleep midpoint from 2:30 AM to 6:30 AM	1.09(1.03,1.16)	< 0.01	1.09(1.03,1.15)	< 0.01	1.07(1.00,1.15)	0.04
P value for nonlinear	0.01		0.01		0.05	

Model 1 was adjusted for age, sex, and race/ethnicity.

Model 2 was adjusted for age, sex, race/ethnicity, education levels, family poverty, marital and smoking status, and alcohol consumption.

Model 3 was adjusted for the variables in model 2 plus hypertension, diabetes, coronary artery disease, stroke, hyperlipidemia, hyperuricemia, waist circumference, BMI, HEI-2015, sedentary time, sleep disturbance.

Abbreviations: OR, odds ratio; CI, confidence interval; BMI, body mass index; HEI, healthy eating index.

Supplement Figure 1. Characteristics of the Missing Values of the Covariates.

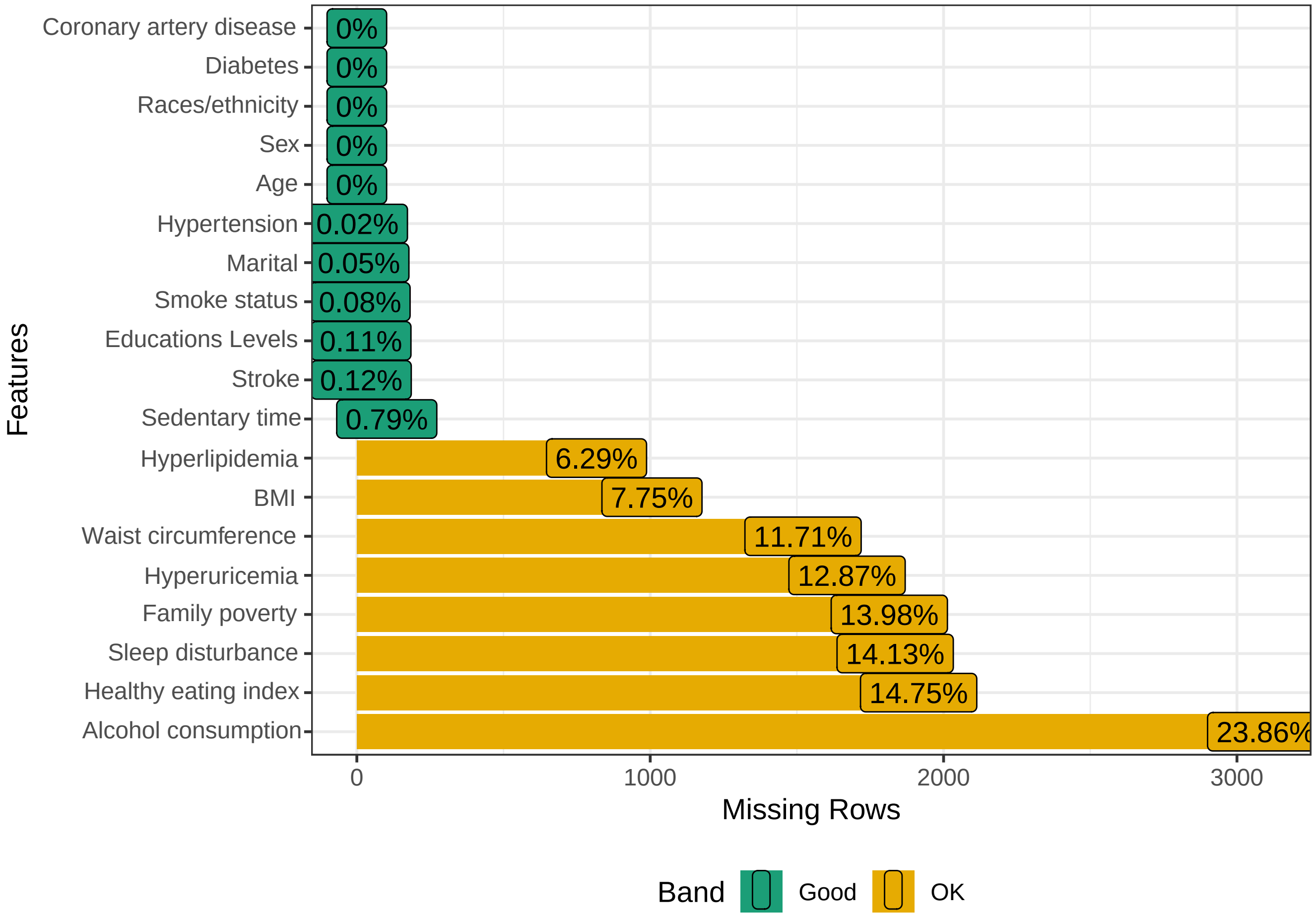

Abbreviations: CI, confidence interval; BMI, body mass index;

Supplement Figure 2. Jitter Diagram of Propensity Score Matching.

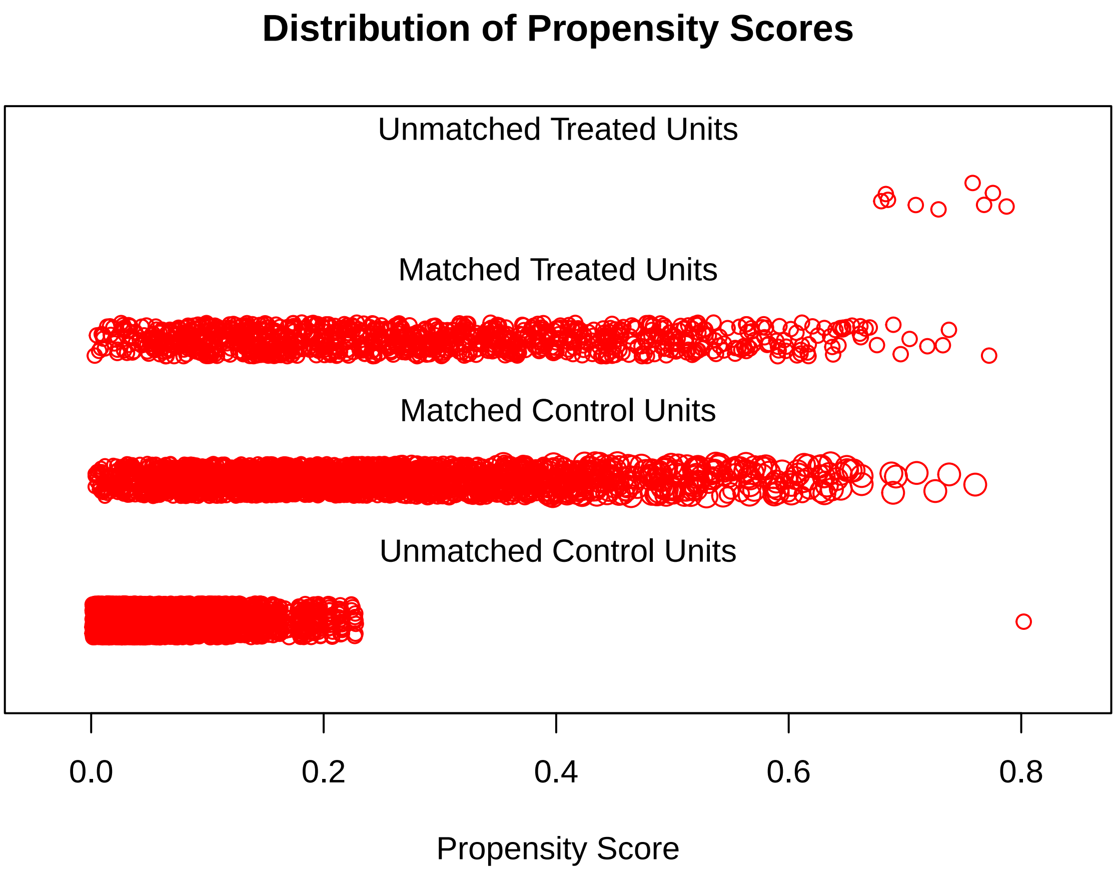

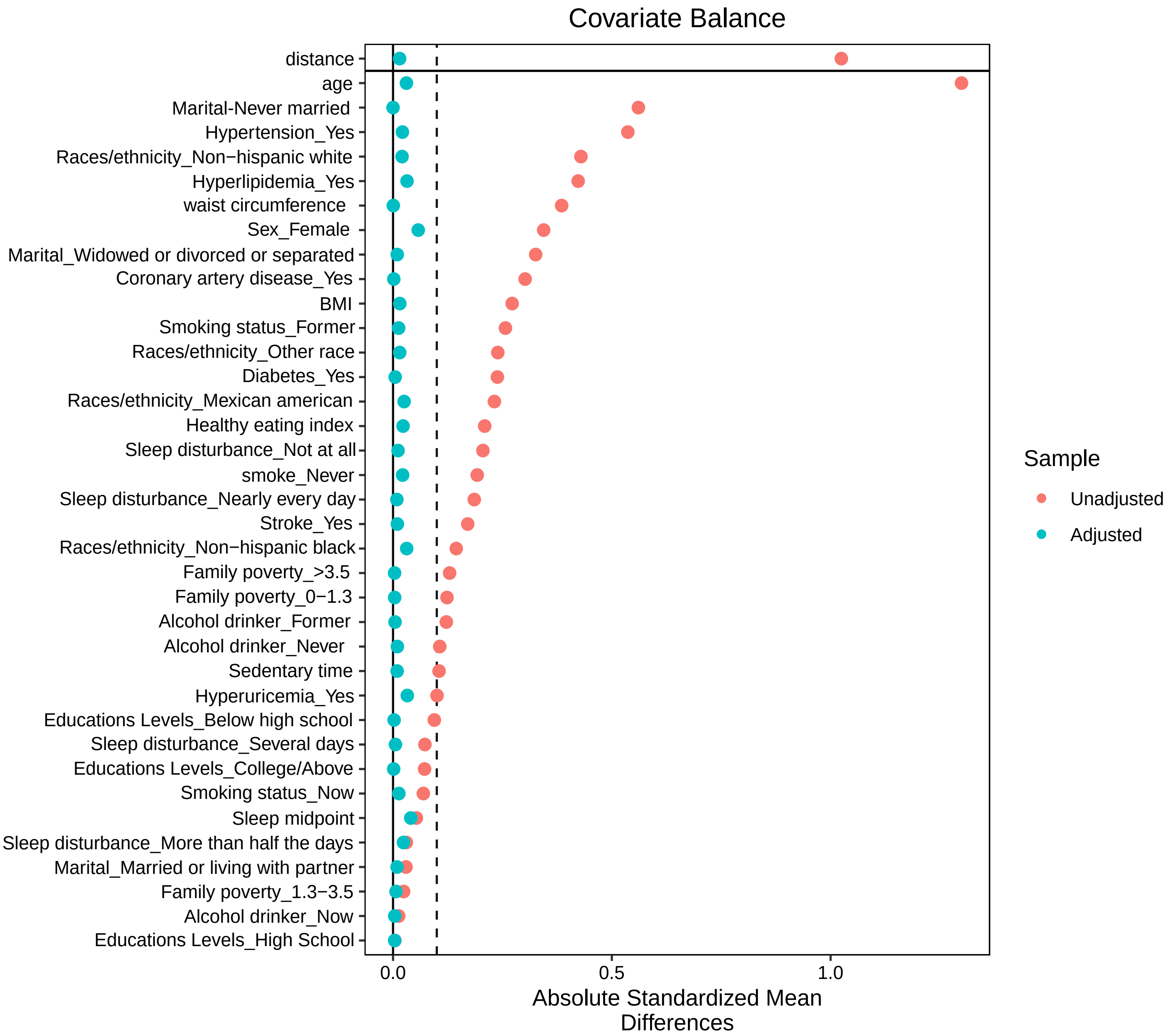

Before undergoing propensity score matching (PSM) at a 1:3 ratio, unmatched treated units, and unmatched non-treated units represented the distribution of individuals in the Osteoarthritis disease (OA)and non-OA groups, respectively. After matching, the two groups were represented by matched treated units and matched non-treated units, respectively. The figure illustrates the balanced distribution of individuals in the matched groups following PSM.

Abbreviations: N, number; BMI, body mass index.
